# Validation of a COVID-19 mental health and wellness survey questionnaire

**DOI:** 10.1186/s12889-022-13825-2

**Published:** 2022-08-08

**Authors:** Maha El Tantawi, Morenike Oluwatoyin Folayan, Annie Lu Nguyen, Nourhan M. Aly, Oliver Ezechi, Benjamin S. C. Uzochukwu, Oluwatoyin Adedoyin Alaba, Brandon Brown

**Affiliations:** 1 Department of Pediatric Dentistry and Dental Public Health, Faculty of Dentistry, Alexandria University, Champolion St., Azarita, Alexandria, 21521 Egypt; 2 Department of Child Dental Health, Obafemi Awolowo University, Ile-Ife, Nigeria; 3 Department of Family Medicine, Keck School of Medicine, University of Southern California, Los Angeles, CA USA; 4 Clinical Sciences Department, Nigerian Institute of Medical Research, Yaba Lagos, Nigeria; 5 Department of Community Medicine, University of Nigeria, Nsukka, Nigeria; 6 Institute of Public Health, Obafemi Awolowo University, Ile-Ife, Nigeria; 7 Department of Employment and Labor Relations, Indiana University of Pennsylvania, Indiana, PA USA; 8 Center for Healthy Communities, Department of Social Medicine, Population and Public Health, University of California, Riverside School of Medicine, Riverside, USA

**Keywords:** Validity, Reliability, Questionnaire, COVID-19, Stress, Multiple correspondence analysis

## Abstract

**Background and aim:**

COVID-19 affected mental health and wellbeing. Research is needed to assess its impact using validated tools. The study assessed the content validity, reliability and dimensionality of a multidimensional tool for assessing the mental health and wellbeing of adults.

**Methods:**

An online questionnaire collected data in the second half of 2020 from adults in different countries. The questionnaire included nine sections assessing: COVID-19 experience and sociodemographic profile; health and memory; pandemic stress (pandemic stress index, PSI); financial and lifestyle impact; social support; post-traumatic stress disorder (PTSD); coping strategies; self-care and HIV profile over 57 questions. Content validity was assessed (content validity index, CVI) and participants evaluated the test-retest reliability (Kappa statistic and intra-class correlation coefficient, ICC). Internal consistency of scales was assessed (Cronbach α). The dimensionality of the PSI sections and self-care strategies was assessed by multiple correspondence analysis (MCA) using all responses and SPSS. For qualitative validation, we used a semi-structured interview and NVivo was used for coding and thematic analysis.

**Results:**

The overall CVI = 0.83 with lower values for the memory items. Cronbach α for the memory items = 0.94 and ICC = 0.71. Cronbach α for PTSD items was 0.93 and ICC = 0.89. Test-retest scores varied by section. The 2-dimensions solution of MCA for the PSI behavior section explained 33.6% (precautionary measures dimension), 11.4% (response to impact dimension) and overall variance = 45%. The 2-dimensions of the PSI psychosocial impact explained 23.5% (psychosocial impact of the pandemic dimension), 8.3% (psychosocial impact of the precautionary measures of the pandemic dimension) and overall variance = 31.8%. The 2-dimensions of self-care explained 32.9% (dimension of self-care strategies by people who prefer to stay at home and avoid others), 9% (dimension of self-care strategies by outward-going people) and overall variance = 41.9%. Qualitative analysis showed that participants agreed that the multidimensional assessment assessed the effect of the pandemic and that it was better suited to the well-educated.

**Conclusion:**

The questionnaire has good content validity and can be used to assess the impact of the pandemic in cross-sectional studies especially as individual items. The PSI and self-care strategies need revision to ensure the inclusion of items with strong discrimination.

**Supplementary Information:**

The online version contains supplementary material available at 10.1186/s12889-022-13825-2.

## Background

Coronavirus infectious disease − 19 (COVID-19), caused by the severe respiratory corona virus-2, was first described in Wuhan, China, in December 2019. It has caused a global pandemic with 481,273,417 million infections and 6,146,034 million deaths as of 27th of March 2022 [1]. For pandemic control, people have been encouraged to take on measures such as physical distancing, respiratory hygiene practices, hand washing and public use of face masks [2]. Many governments have also adopted strict public health measures like quarantine, isolations, and total or partial lockdowns resulting in restricted human movements and limited physical socialisation [3]. Additionally, various vaccines are being rolled out by countries to protect against infection.

The pandemic and the necessary measures taken to control the pandemic had unintended negative impact on the economic, medical condition, mental health and wellbeing of individuals. It caused stress, anxiety and depression resulting from concerns about loss of health, life, wealth, and economic opportunities [4]. Concerns about the unknown impact of the pandemic on the future made it difficult to plan [5] and the limited access to social support due to physical and social distancing became sources of stress [6]. Loss of job, income and health insurance during the pandemic may have exacerbated existing barriers to health care and reduced access to care [7, 8]. For those in low resource settings, the majority had no health insurance, and there were challenges to physically access health care facilities during the lockdown. In addition, low prioritisation of non-COVID related healthcare services may have worsened the overall health of citizens [9]. The disruption of essential services for HIV, tuberculosis, malaria and other childhood diseases, is estimated to have caused huge reversals in gains made over the last several years [10].

Stress induced concerns related to anxiety, changes in concentration, insomnia, interpersonal conflicts, irritability, and reduced productivity [5]. Post-traumatic stress disorder may result from being infected with COVID-19, stigma related to being COVID-19 infected, fear of exclusion, social isolation associated with public health containment measures; and witnessing the suffering related to the COVID-19 pandemic [11].

Despite the feelings of anxiety, stress and disruptions to life caused by the COVID-19 pandemic, people cope with challenges [12]. However, the ability to adapt is dependent on multiple mutable and immutable factors such as genetic background, access to healthcare, literacy, living conditions, life experiences, poverty, employment status, and access to social support [13]. Having pre-existing medical conditions may also worsen the ability to adapt to the challenges of COVID-19 infection [14].

Because COVID-19 is a novel disease, there is a need for empirical data on the impact of the COVID-19 pandemic on the mental health and wellbeing of individuals and there is an urgent need for researchers to collect data in real time. Standardised and validated instruments facilitate comparative studies across countries and regions of the world. This helps in the design of interventions to address the pandemic impact at country, regional and international levels. Policy makers can use this and similar multidimensional assessments of various aspects of the pandemic impact to design support measures and packages tailored to the background of different subgroups taking into consideration the relative importance of different factors as the pandemic unfolds. Previous studies reported the development of tools assessing the impact of COVID-19 on mental health including fear [15], anxiety [16, 17], stigma [18], and psychosocial reactions [19].

The design of the questionnaire acknowledges that negative information received from media may be amplified by the environment created by the response to the COVID-19 pandemic resulting in negative emotions and stress [20]. Physical and social distancing, quarantine and isolation impact negatively on mental health and increases the risk of mortality [21]. Also, there are possible socioeconomic effects on behaviour associated with the pandemic that need to be studied to understand how the socioeconomic status of individuals can impact on the behaviour of different populations during the pandemic [22] with possible differences by income profiles of countries and regions of the world.

These multiple effects of the pandemic call for multidimensional assessment of its impact on the health and wellness of individuals. We describe how we developed and validated a multidimensional assessment method named the COVID-19 mental health and wellness (MEHEWE) questionnaire. This multidimensional assessment can be used to collect data to determine the relationships between COVID-19 related variables and the health and wellbeing of respondents.

## Methods

### Study design

We used a mixed methods approach for validation. We developed multidimensional assessment to collect details on the sociodemographic and medical health profile of respondents, measure the pandemic stress level, identify the impact of COVID-19 pandemic on the finance and lifestyle of respondents and identify the level of perceived social support by respondents. This was adapted from an instrument initially developed by a team of researchers including two of the present study team (ALN and BB) to collect data on the effect of the COVID-19 pandemic on people living with HIV (PLHIV) in the United States [23] due to the similarity between the COVID-19 pandemic situation and the early time of AIDS. The current study tested the multidimensional assessment for content validity, dimensionality and reliability on an international audience. In-depth interviews were also conducted with a sample of respondents to discuss their perception about the comprehensiveness of the multidimensional assessment in capturing their COVID-19 experience.

### Survey instrument (supplementary file 1)

SECTION 1- Sociodemographic profile (16 questions): This section had 11 questions about age, sex at birth, country of residence, current gender, highest level of education attained (none, primary, secondary, university, postgraduate), employment status, health insurance status, marital status, person whom respondent currently resides with, sexual orientation and sexual practices. Respondents could check one or more of these options. This section also included 5 questions assessing COVID-19 experience on a Yes/ No basis: whether the respondent tested positive for COVID-19 infection, was suspected but not tested, had close friends who tested positive, knew someone who died of COVID-19 infection or had to isolate because of COVID-19 infection.

SECTION 2- Medical health profile (2 questions): Respondents selected the conditions they had from a checklist of 28 health conditions in addition to other health conditions not on the checklist. This checklist was adapted from the study by Marg et al. [24] and included infectious diseases (hepatitis, herpes, pneumonia, shingles, and sexually transmitted disease), non-infectious diseases (diabetes, cancer, dermatologic problems, heart condition, hypertension, migraine, neurological problems, neuropathy, respiratory problems, and stroke), and geriatric conditions (arthritis, broken bones, depression, loss of hearing and vision). The section also assessed subjective cognitive state and memory based on the MAC-Q developed by Crook et al. [25]. It consisted of 5 questions and a global statement rated on a 5-point likert scale and a sixth global statement. The total score is the sum of the scores of the sixth statement with double weight for the sixth statement. The total score ranges from 7 to 35 with higher scores indicating greater impairment. Scores above 25 indicate memory loss [25].

SECTION 3- Pandemic stress level (6 questions): The Pandemic Stress Index (PSI) is a 3-section measure of behavior changes, impact on daily lives and stress the individual may have experienced during the COVID-19 pandemic [26]. The tool has not been previously validated. Authors of the original PSI recommend adding population-specific items as necessary depending on study or clinical needs. As such, several modifications were made for this study. The first section assessed behavior changes in response to the COVID-19 experience including 12 changes related to public health messages (physical distancing, isolation, quarantine), the workplace (working remotely, job loss), and to protect one’s own or others’ health (caretaking). Three additional questions assessed details of changes in work status, travel plans and use of healthcare services. The second section rated the impact of the COVID-19 experience on daily life on a 5-point scale. The third section assessed the psychosocial impact of the COVID-19 experience including 20 items related to emotional distress, and difficulties faced because of the pandemic such as change in work status, use of healthcare services and travel plans [27].

SECTION 4- Finance and lifestyle impact (12 questions): This section assessed changes in lifestyle using a question of seven items including sexual activity, use of tobacco, alcohol, marijuana, other substances, food intake, and use of screens The response to these items was either increase, decrease, or no change. There was a second question of 10 items: seven assessing the impact of the COVID-19 pandemic on the finance of respondents, and three items assessing the impact on access to food and meals. Responses to these questions were either ‘yes’ or ‘no’. The questions on finances were adopted from the Multi-center AIDS Cohort Study (MACS)/Women’s Interagency HIV Study (WIHS) Combined Cohort Study questionnaire [28], while those on food and meals were adapted from the US Department of Agriculture Household Food Security Survey [29].

This section also included ten questions assessing further details on the impact on financial condition, critical medical care, access to healthcare, alternative treatment, access to mental care, substance abuse care, ability to keep healthcare provider appointment adapted from the MACS/WHIS questionnaire [28].

SECTION 5- Perceived level of social support (6 questions): There were two questions assessing respondents’ feeling of isolation (on a scale of 1 -not at all – to 10 – extremely) and perception of how the pandemic affected their sense of isolation. These were developed by the study team. There were four other questions about the difficulty of adhering to social distancing, and changes in the quality of relationship with family, friends and significant others on a 5 points likert scale. These were adopted from the Coronavirus Health Impact Survey (CRISIS) Adult Self-Report Baseline questionnaire [30].

SECTION 6- Post-traumatic stress disorder (one question): This included a question of 17 items adopted from the post-traumatic stress disorder (PTSD) Checklist – Civilian Version (PCL-C) [31, 32]; a standardized self-reported rating scale assessing symptoms of post-traumatic stress. The responses were on a 5-point likert-like scale ranging from not at all (scored 1), to extremely (scored 5) with a total score which is the sum of items’ scores ranging from 17 to 85. Scores from 17 to 29 indicate no severity, from 28 to 29 indicate some severity, from 30 to 44 indicate moderate to moderately high severity and 45 and above indicate high severity of PTSD symptoms [31]. Its diagnostic accuracy as a screening tool needed assessment for population and context specific validation [33].

SECTION 7- Coping strategies (one question): This section was a grid of three items adopted from the Brief Resilient Coping Scale [34]. Responses were on a 5-point likert-like scale ranging from does not describe me at all (scored 1), to describes me very well (scored 5). The total was the sum of scores ranging from 3 to 15. Scores of 3–8 indicate low resilient coping, 9–11 indicate medium resilient coping and 12–15 indicate high resilient coping.

SECTION 8- Self-care (three questions): There was a checklist of eleven items assessing what the respondents did to take care of their mental health during the COVID-19 pandemic. The items included talking with family and friends through phone, video chats, and/or in-person, spending time with pets, meditation, exercise in-doors and out-doors, gardening and hobbies, learning new skills and/or taking breaks from news in addition to an option to specify other activities not in the list. There were also open-ended questions to list challenges faced during the COVID-19 pandemic and other strengths or resiliencies tapped into that the multidimensional assessment did not capture. This section was internally developed.

SECTION 9- For PLHIV (nine questions): This section included standard questions about HIV clinical characteristics such as year of diagnosis and last viral load and CD4 count, status of current HIV medication refill, missing doses of HIV medications during the pandemic, and reasons for missed doses. Reasons for missed doses of HIV medications were adapted from the AIDS Clinical Trial Group instrument for missing medications [35].

### Study procedures

Data was collected using Survey Monkey®, an online survey platform. The links to the survey were prepared with settings to ensure that it would be anonymous, that participants could change their answers freely before they choose to submit, and it was not time-limited. One submission per electronic device was allowed. We created the questionnaire in English, and translated it to four other languages (Arabic, French, Portuguese and Spanish) followed by back translation to English by bilingual speakers to ensure accuracy based on the World Health Organization recommendations [36]. Links were sent to eligible participants – persons who were 18 years and older, could give consent, and could read the survey in any of the available languages- through emails and social media platforms. The survey was opened on the 29th of June 2020 and closed on the 31st of December 2020.

### Quantitative validation of survey instrument

Step 1: Completeness of the paper-based version. Seven members of the study team (core team) reviewed a draft of the questionnaire to assess comprehensiveness in capturing all the elements of mental health and wellbeing that the study addressed; that the sequence of the questions was logical; that the questions were culturally appropriate and that they would not breach any ethical concerns. The review was conducted between 18th and 25th of May 2020. At the end of this step, the list of medical conditions in section 2 was updated following the cited reference, a question about health insurance was added in section 2 and two items were added to the self-care strategy in section 8.

Step 2: Clarity of electronic format. Based on the feedback obtained in step 1, a revised questionnaire was converted to an electronic format. The seven core team members and seven other invited collaborators responded to the online survey and timed themselves to calculate the time taken to respond to the survey and to check clarity. The review took place between 28th of May and 3rd of June 2020. Thirty-four unique comments were received from nine of the 14 evaluators.

Step 3: Content validity. Using comments from the previous steps, the revised questionnaire was launched on the 5th to the 10th of June 2020 and thirteen collaborators were invited to take the survey and fill out a form in which they evaluated each item in the survey on a 4-point scale ranging from least (scored 1) to most (scored 4) relevant to assess content validity, calculate the content validity index (CVI) and make sure that per item and overall values were at least 0.78 [37].

Step 4: Test-retest reliability. 350 respondents of the final survey were invited within a week of completion of the questionnaire the first time to refill the questionnaire a second time. These were respondent who agreed to be contacted again about the study. The final number of respondents included in the assessment of the test-retest reliability was 227 (64.9%).

### Qualitative validation of survey instrument

A qualitative exploration of validity, specificity, and sensitivity of the multidimensional assessment was conducted in English. The qualitative validation was modelled against that conducted by Engel et al. [38]. A semi-structured topic guide was used with open-ended questions supplemented with probes, where necessary, to assess participants’ emerging accounts and perspectives. Participants were asked to reflect on their understanding of mental health and wellness, factors influencing it, and the possible impact of the COVID-19 pandemic on it. Next, a cognitive debriefing exercise was conducted to assess the face validity of the multidimensional assessment. Participants were provided with copies of the questionnaire and asked to explore whether sections with questions and response options were appropriate and acceptable, interpreted accurately and relevant to participants’ lived experiences. Participants were asked the following questions: (1) What were your immediate thoughts about this questionnaire? (2) Was the wording of questions and response options clear? (3) Do you think the questionnaire was applicable to people affected by the COVID-19 pandemic (4) Was it comprehensive? (5) Were there any aspects you think were missing? The interview explored respondents’ observations on practical and interpretative problems they experienced in completing the survey including time spent filling the questionnaire, challenges with filling it, motivation to fill it, what may influence variability in responding to the same question, and suggestions for further modification of the questionnaire.

For similar qualitative interviews, saturation was reached with a sample of 12 persons when working with a homogeneous group [39]. The sample for qualitative assessment was drawn from the respondents who participated in the test-retest instrument reliability assessment. This meant the respondents have been exposed to the questionnaire at least twice within two weeks. Participants who were involved in the test-retest survey were asked to volunteer for the qualitative interviews by sharing their emails for further contact. The volunteers were contacted through their emails and were scheduled for either a phone interview or a teleconference interview based on their preference. Respondents were provided with soft copies of the questionnaire ahead of the interview. Those who opted for a Zoom interview were reimbursed $2.78 (N1000) for their data. Phone interviewees did not get reimbursed since the interviewer paid for the call.

### Ethical consideration

The study protocol was submitted to Institute of Public Health Research Ethics Committees, Obafemi Awolowo University Ile-Ife, Nigeria (IPHOAU/12/1557). Additional ethical approvals were attained from India (D-1791-uz and D-1790-uz), Saudi Arabia (CODJU-2006F), Brazil (CAAE N° 38,423,820.2.0000.0010) and the United Kingdom (13,283/10570). Participants were required to provide informed consent before filling the online questionnaire. All data were irrevocably anonymised. We took measures to prevent the unintended collection of IP to protect the privacy of participants and the confidentiality of the information they provided. IP addresses were instantly decoupled from the questionnaire, encrypted, and deleted at the end of the online survey by the survey tool. The questionnaire also did not install any tracker cookies on the device of the respondents. The study was made available to the target population through a secure, SSL encrypted connection link. Data in transit (while responding online) were encrypted using secure TLS cryptographic protocols. The collection tool was certified in compliance with the EU-U.S. Privacy Shield Framework and Swiss-U.S. Privacy Shield. Due to the anonymity given to participants and the IP addresses being decoupled and encrypted automatically during the time that the survey was online, there was no possibility to provide further direct information to participants after completion of the questionnaire.

### Data analysis

Quantitative analysis: For test-retest reliability of the categorical responses, the Kappa statistic was calculated. When a score was obtained by adding the scores of individual items, we assessed the internal consistency of the items by calculating Cronbach’s α then calculated the intra class correlation coefficient (ICC) of the overall score. We modified the Landis and Koch categorisation and classified Cronbach’s α, ICC and Kappa statistic for reliability and/ or internal consistency into 0–0.39 (low level), 0.40–0.79 (moderate level) and 0.81–1 (excellent level) [40].

To assess the dimensionality, structure and relationships between the various items in the PSI behavior change section and psychosocial impact section as well as the items of the self-care strategies, we used multiple correspondence analysis (MCA) of responses from the whole sample. These items were categorical variables for which the use of MCA is suitable. MCA is a data reduction technique similar to principal component analysis that can be used with nominal variables [41]. MCA can reveal patterns by representing levels of variables as points in a cloud overlayed on a Euclidean space. The points are plotted on X by Y axes for a 2-dimensions solution. The proximity of these points to each other and their distribution along the X and Y axes describe emerging subgroups in a set of variables.

We used the variable principal normalization method to obtain a two-dimensions solution to enable data interpretation [42]. We calculated the discrimination measures and produced a joint plot (biplot) of category points. This is a type of scatter plot to identify category relationships and subgroup membership. Points (representing levels of variables) in the same quadrant belong to the same subgroup. We also displayed the discrimination measures plot where the length and steepness of lines indicate the importance of the variables. Variables with unique characteristics are located far from others and from the origin [43]. The data was analysed using IBM SPSS Statistics version 23.0.

Qualitative analysis: the transcribed interviews were imported into Nvivo 11 to facilitate data coding, retrieval [44] and thematic analysis [45]. Thematic analysis consisted of the following stages: familiarisation with the data (reading the transcripts); generating initial codes (organizing data into meaningful groups); searching for themes (sorting the codes into potential themes); reviewing themes (refining themes); defining and naming themes (development of a thematic map of the data and description of the content of each theme) [45]. The thematic analysis identified domains that were perceived as relevant to the focus of the survey. These domains were then compared with the content of the survey. Also, the comments made by the participants for the nine sections of the survey were summarised.

## Results

### Content validity

Table 1 shows that the CVI for each section was ≥0.79 except for section 2 assessing medical health profile and memory (CVI = 0.71) where the CVI for items assessing the medical condition was 0.92 whereas the CVI of the MAC-Q items was 0.68. The overall CVI of the whole questionnaire was 0.83.

**Table 1:** Content validity index of the different sections of the survey assessed by 13 experts

Section #	Section label	CVI
1	Sociodemographic profile and COVID experience	0.83
2	Medical health status	0.71
3	Pandemic stress index	0.90
4	Financial and lifestyle	0.90
5	Psychosocial support	0.90
6	PCL-C	0.81
7	Coping score	0.90
8	Self-care strategies	0.97
9	For people living with HIV	0.79

### Test-retest reliability

A total of 227 participants, mostly from Nigeria, were included in the test-retest reliability assessment. The survey took about 13.5 minutes to complete. The second set of responses was obtained within a mean (SD) of 17.3 (7.0) days after the first response. Table 2 shows that most participants (59%) were females, and their mean (SD) age was 40.1 (11.0) years. Most participants had a postgraduate degree (52%), were employed full time (54.6%), had no health insurance (50.2%) and were living with their partners (46.3%).

**Table 2:** Sociodemographic profile of participants in the test-retest reliability assessment [*N* = 227]

Characteristics		Number (%)
Sex at birth	Male	93 (41)
	Female	134 (59)
Age (years)	Mean (SD)	40.1 (11.0)
Country	Nigeria	162 (71.4)
	Others^a^	65 (26.6)
Education	Secondary	14 (6.2)
	University	95 (41.9)
	Postgraduate	118 (52)
Employment status	Employed full time	124 (54.6)
	Employed part time	27 (11.9)
	Unemployed	34 (15.0)
	Self-employed, retired or student	42 (18.5)
Health insurance	No	114 (50.2)
	Yes	113 (49.8)
Living conditions	With spouse or partner	105 (46.3)
	Living with other family members	73 (32.2)
	Alone or with other people	49 (21.6)

^a^Other countries include Zimbabwe, USA, India, Bosnia and Herzegovina, Canada, UK, Saudi Arabia, Brazil, Mauritius, Netherlands, Peru, South Africa, Thailand, Uganda, Australia, Belgium, Egypt, Finland, Ghana, Indonesia, Mexico, Philippines, Sierre Leone, Turkey, UAE

Table 3 shows the test-retest reliability and internal consistency statistics. The minimum Kappa statistic in sections 2–5 and 8 was less than 0.4 indicating low test-retest reliability in these sections. The minimum Kappa in sections 1 and 9 was > 0.4 and < 0.8 indicating moderate test-retest reliability.

**Table 3:** Test-retest reliability and internal consistency statistics

#	Section label		Kappa	ICC	Cronbach α
1	Sociodemographic profile and COVID experience information		0.48–0.99		
2	Medical health status	Medical conditions	0–1.00		
		Memory condition (MAC-Q)	–	0.71	0.94
3	Pandemic stress index	Behavior changes	0–0.66		
		Perceived impact	0.32		
		Psychosocial effects	0.09–0.91		
4	Financial and lifestyle		0.26–0.83		
5	Psychosocial support	Perceived social isolation	–	0.65	
		Isolation compared to before the COVID pandemic and perceived difficulty of adhering to measures	0.24–0.41		
		Impact on relations with family, significant others and friends	0.26–0.42	0.30	0.70
6	PCL-C			0.89	0.93
7	Coping			0.69	0.56
8	Self-care		0.25–0.73		
9	For people living with HIV		0.57–1.00		

The ICC for test reliability of sections 6 items was 0.89 indicating excellent reliability. The ICC values for the MAC-Q items, the perceived social isolation, and section 7 items were 0.71, 0.65, and 0.69 indicating moderate reliability whereas the ICC for the impact on relations items was 0.30 indicating low reliability.

The internal consistency of MAC-Q and the PCL-C items were excellent (Cronbach α = 0.94 and 0.92) and the α of values of the impact on relations and coping score were lower (0.70 and 0.56) indicating less internal consistency.

### Dimensionality

Responses were available from 21,106 participants from 152 countries (Supplementary file 2). There were > 200 participants from each of Nigeria, Pakistan, Saudi Arabia, India, Egypt, USA, UK, Jordan, Mexico, South Africa, Argentina, Syria, Philippines, Finland, Ghana, Yemen, Bosnia and Herzegovina, Sudan and Hungary. Table 4 shows that most participants were females (53.3%) aged 34.9 years on average. Most participants were university educated (42.4%) or higher (25.4%) and self-employed, retired or students (33.3%) with health insurance (43.4%) and living with other family members (43.4%).

**Table 4:** Sociodemographic profile of participants included in the MCA of the PSI and self-care strategies [*N* = 21,106]

Characteristics		Number (%)
Sex at birth	Male	6853 (32.5)
	Female	11,249 (53.3)
	No response	3004 (14.2)
Age (years)	Mean (SD)	34.9 (12.9)
Education	Less than secondary education	742 (3.5)
	Secondary	3214 (15.2)
	University	8946 (42.4)
	Postgraduate	5351 (25.4)
	No response	2853 (13.5)
Employment status	Employed full time	6900 (32.7)
	Employed part time	1529 (7.2)
	Unemployed	2006 (9.5)
	Self-employed, retired or student	7029 (33.3)
	Home carer	789 (3.7)
	No response	2853 (13.5)
Health insurance	No	9082 (43.0)
	Yes	9171 (43.4)
	No response	2853 (13.5)
Living conditions	With spouse or partner	6312 (29.9)
	Living with other family members	9142 (43.3)
	Alone or with others people	3390 (16.1)

**Fig. 1:**
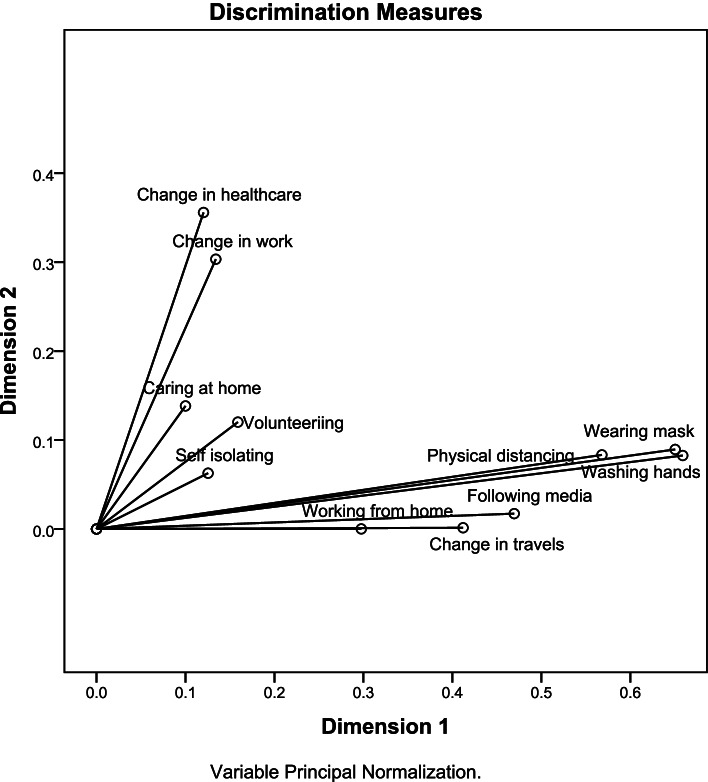
Discrimination measures of the MCA for behavior change during the pandemic in the PSI

Table 5 shows the 2- dimensions MCA solution of behavior changes during the pandemic in the PSI. The % of variance explained by dimension 1 was 33.6% and by dimension 2 was 11.4% with mean variance explained = 22.5% and overall variance explained by the two dimensions = 45%. Table 5 and Fig. 1 show that the most discriminant behaviors for dimension 1 in descending order were washing hands, wearing masks and practicing physical distancing (discriminant measures = 0.66, 0.65 and 0.57). In dimension 2, the discriminant measures had lower values and the strongest were change in health care services use and change in work status (discriminant measures = 0.36 and 0.30). Some measures had weak mean effect and weak discrimination in dimension 1and 2 such as self-isolation (discriminant measures mean = 0.09, in dimension 1 = 0.13 and in dimension 2 = 0.06). Dimension 1 includes behaviors to protect against the pandemic (precautionary measures) and dimension 2 reflects the impact of the pandemic (impact of the pandemic). The internal consistency of dimension 1 was much higher than that of dimension 2 (Cronbach α = 0.80 and 0.22).

**Table 5:** Discrimination measures of changed behaviour during the pandemic in the PSI [*n* = 21,106]

Variables	Dimension 1 Precautionary measures	Dimension 2 Impact of the pandemic	Mean
Practicing physical distancing	0.57	0.08	0.33
Self-isolating	0.13	0.06	0.09
Wearing masks	0.65	0.09	0.37
Washing hands	0.66	0.08	0.37
Caring for someone at home	0.10	0.14	0.12
Working from home	0.30	0.00	0.15
Volunteering	0.16	0.12	0.14
Following media for pandemic coverage	0.47	0.02	0.24
Change in work status	0.13	0.30	0.22
Change in healthcare services use	0.12	0.36	0.24
Change in travel plans	0.41	0.00	0.21
Total	3.70	1.25	2.48
% of variance	33.6	11.4	22.5
Cronbach α	0.80	0.22	0.66

**Fig. 2:**
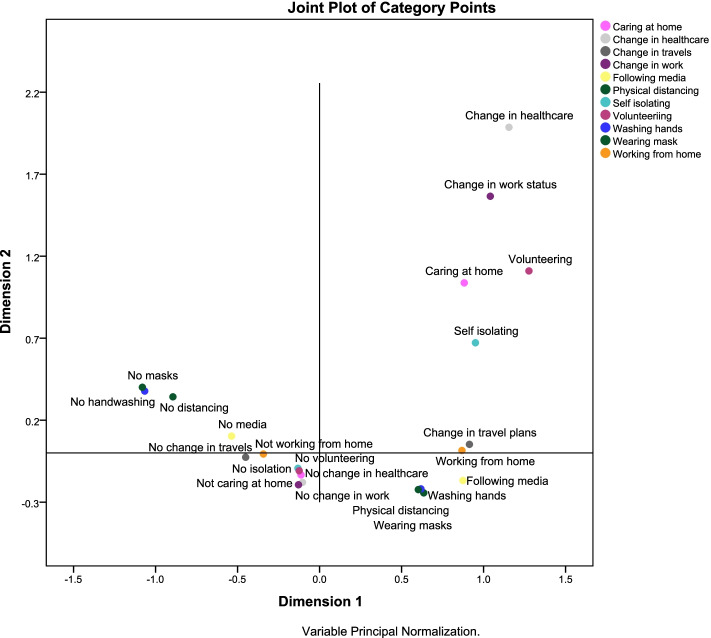
Joint plot of category points for the 2-dimension MCA solution for behavior change during the pandemic in the PSI

Figure 2 shows that the responses discriminated between participants on the two dimensions to 4 subgroups: those who had change in access to healthcare, change in work status, cared for someone at home, self-isolated and volunteering; those who did not isolate, did not care for some at home, had no change in work, and did not volunteer; those who worked from home, washed their hands frequently, practiced physical distancing, and wore masks; and those who did not wear masks, did not wash their hands, did not practice physical distancing, and did not follow media coverage.

**Fig. 3:**
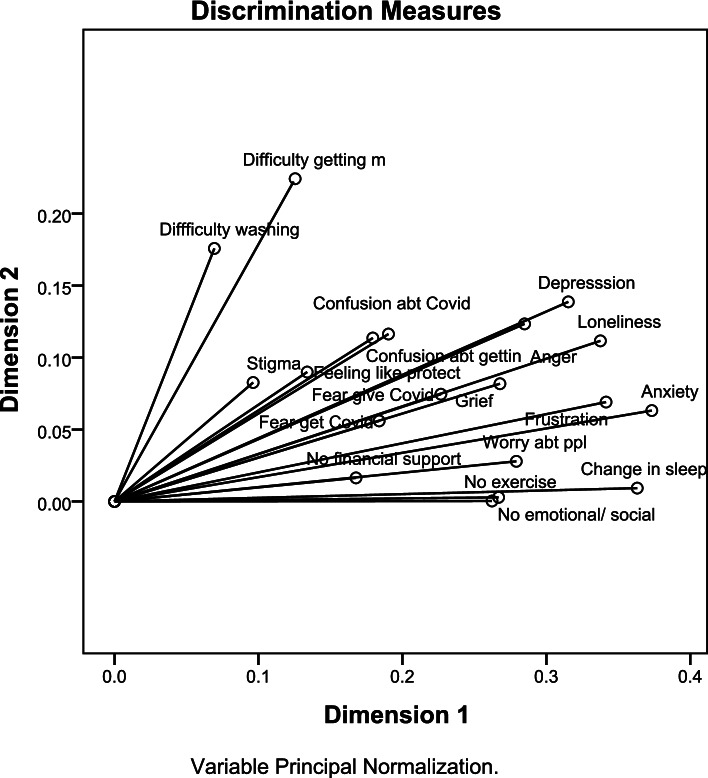
Discrimination measures of the psychosocial impact of the pandemic of PSI

Table 6 and Fig. 3 show the discriminant measures of the 2-dimensions solution for the psychosocial impact of the COVID-19 pandemic of the PSI. Dimension 1 explained 23.5% of variance and dimension 2 explained 8.3% of variance with mean variance explained = 15.9% and overall variance explained by the two dimensions = 31.8%. The strongest discriminant measures in dimension 1 were anxiety, changed sleep, frustration, loneliness and depression (discriminant measures = 0.37, 0.36, 0.34, 0.34 and 0.32). The discriminant measures were much weaker in dimension 2 and the strongest among them were difficulty getting masks and difficulty washing hands (discriminant measures = 0.22 and 0.18). Some discriminant measures had low mean value and low values in each dimension such as stigma (mean discriminant measure = 0.09) and no financial support (mean discriminant measure = 0.09).

**Table 6:** Discrimination measures of psychosocial impact during the pandemic of the PSI [*n* = 21,106]

Variables	Dimension 1 Impact of the pandemic	Dimension 2 Impact of the precautionary measures	Mean
Fear of getting COVID infection	0.19	0.06	0.12
Fear of giving COVID infection	0.23	0.08	0.15
Worrying about people	0.28	0.03	0.15
Stigma	0.10	0.08	0.09
Frustration	0.34	0.07	0.21
Anxiety	0.37	0.06	0.22
Depression	0.32	0.14	0.23
Loneliness	0.34	0.11	0.23
Anger	0.29	0.12	0.20
Grief	0.27	0.08	0.18
Change in sleep	0.36	0.01	0.19
Confusion about what COVID infection is	0.18	0.11	0.15
Feeling like protecting oneself and others	0.13	0.09	0.11
No emotional/ social support	0.27	0.00	0.14
No financial support	0.17	0.02	0.09
No exercise	0.26	0.00	0.13
Confused about getting correct information	0.19	0.12	0.15
Difficulty getting masks	0.13	0.22	0.18
Difficulty washing hands	0.07	0.18	0.12
Total	4.46	1.58	3.02
% of Variance	23.5	8.3	15.9
Cronbach α	0.82	0.39	0.71

Dimension 1 includes the impact of the pandemic itself and dimension 2 includes the impact of the precautionary measures of the pandemic. Dimension 2 explained a low percentage of variance and some of its discrimination measures had higher discrimination effect in dimension 1 including depression and anger. The internal consistency of dimension 1 was much higher than dimension 2 (Cronbach α = 0.82 and 0.39).

**Fig. 4:**
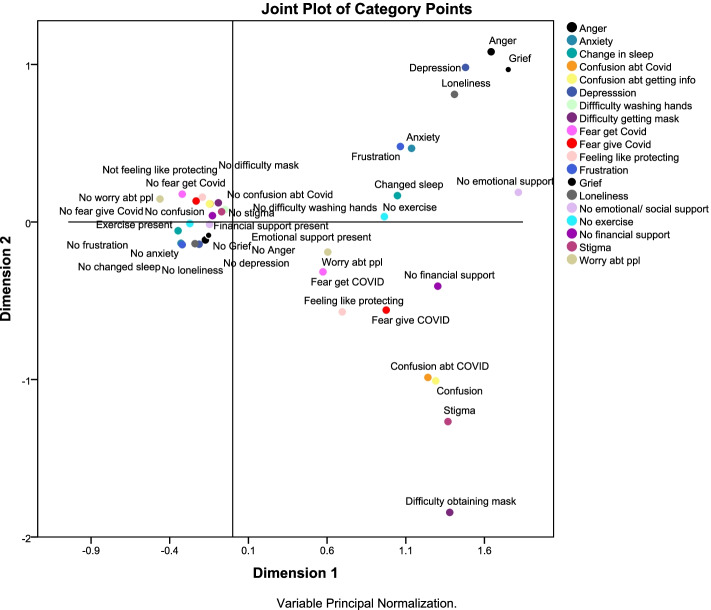
Joint plot of category points for the 2-dimension solution of the psychosocial impact of the pandemic of the PSI

Figure 4 shows that the two dimensions discriminated between participants into four subgroups: those who felt anger, grief, depression, loneliness, anxiety, frustration and no emotional support; those who felt no frustration, no loneliness, no change in sleep pattern, and no anxiety; those who felt difficulty obtaining masks, felt stigma, confusion, fear of giving COVID-19 infection, felt the need to protect others, worried about people and felt no financial support; and those who did not feel like protecting others, did not have fear of getting COVID-19 infection, did not worry about people, and did not fear giving COVID-19 infection to others.

**Fig. 5:**
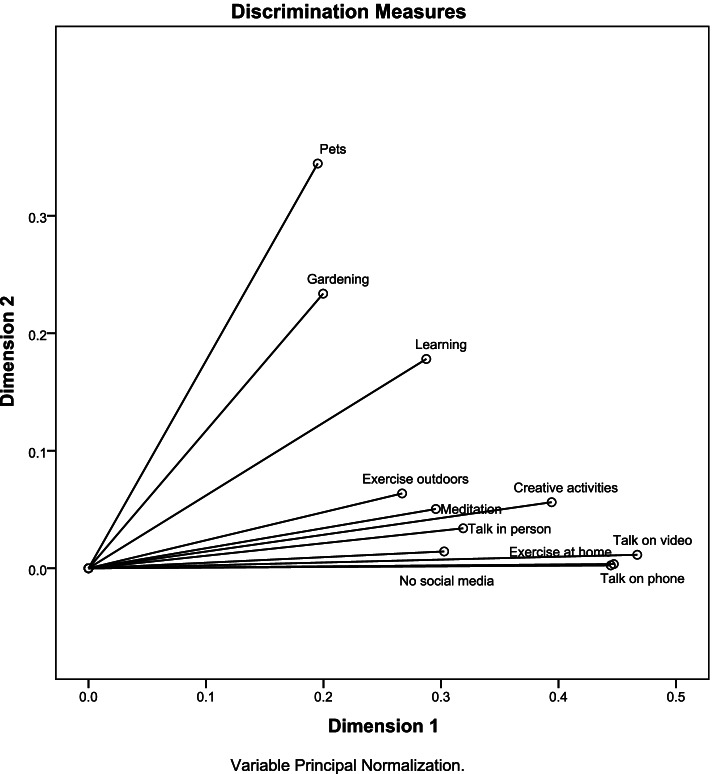
Discrimination measures of the self-care strategies during the pandemic

Table 7 and Fig. 5 show the 2-dimension solution for the self-care strategies used during the pandemic. The discrimination measures in dimension 1 explained 32.9% of variance and those in dimension 2 explained 9.0% with mean variance = 21% and overall variance explained by the two dimensions = 41.9%. In dimension 1, the strongest discrimination measures were talking on video chat, exercising at home, talking on the phone and creative activities (discrimination measures = 0.47, 0.45, 0.44 and 0.40). The strongest measures in dimension 2 were spending time with pets, gardening and learning new things (discrimination measures = 0.34, 0.23 and 0,18). Dimension 1 includes strategies used by people who are not outward going and who may be avoiding contact with others whereas dimension 2 includes strategies by outward-going people. The internal consistency of dimension 2 was very low (Cronbach α- 0.01).

**Table 7:** Discrimination measures of self-care strategies during the pandemic [*n* = 21,106]

	Dimension 1 Inward activities	Dimension 2 Outward activities	Mean
Talk on phone	0.44	0.00	0.22
Talk on video	0.47	0.01	0.24
Talk in person	0.32	0.03	0.18
Pets	0.20	0.34	0.27
Meditation	0.30	0.05	0.17
Exercise at home	0.45	0.00	0.23
Exercise outdoors	0.27	0.06	0.17
Gardening	0.20	0.23	0.22
Creative activities	0.39	0.06	0.23
Learning	0.29	0.18	0.23
No social media	0.30	0.01	0.16
Total	3.62	0.99	2.31
% of Variance	32.9	9.0	21.0
Cronbach α	0.80	0.01	0.62

**Fig. 6:**
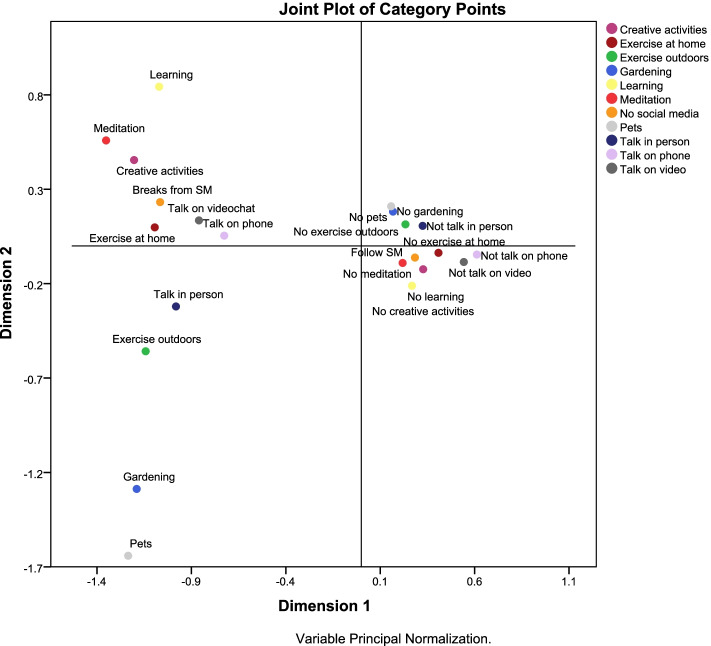
Joint plot of category points for the 2-dimension of the self-care strategies during the pandemic

Figure 6 shows that the two dimensions classify participants into four groups: those who do not exercise outdoors, do not talk in person, do not spend time with pets, do not engage in gardening and do not talk on phone; those who spend time with pets, who garden, who exercise outdoors, and who talk in person; those who learn new things, have creative activities, take breaks from social media, talk on video chat, talk on the phone and exercise at home; and those who do not meditate, follow social media, do not learn new things, have no creative activities, and do not talk on video chat.

### Qualitative findings

All participants acknowledged that the questionnaire assessed the effect of the COVID-19 pandemic. Most respondents believed everyone was affected by the COVID-19 pandemic though the impact varied between individuals and so the survey was applicable to all persons. There was consensus that a study assessing the impact of the pandemic was timely as interventions were needed to help persons who were negatively affected by the pandemic. A respondent noted:

‘Invariably, COVID 19 affected so many people in one way or the other. COVID 19 affected many people mentally. You know, in Nigeria, things are not balanced up, especially those who are living on daily income. If we want to talk about people who are living on daily income, those people that if they don’t go out, they don’t have anything to spend, the effect of that, I think the anxiety they are facing inside their homes will affect them mentally.’

Two respondents felt the survey was more applicable to people with health challenges, especially those who needed to visit health facilities due to their health conditions.

‘The questionnaire was structured in a way that it specifically addresses those who have health challenges during the COVID-19 pandemic. That’s my perception. Because if we look at the content of the questionnaire, you would realize that it is asking information about health status and asked whether you are asthmatic, whether you are hypertensive, whether you are sick, have you been able to see your medical practitioner? And so on. So, it is not really for those who are well, so to say, as in those who are healthy per se.’

All respondents felt the wording of the questionnaire was clear, and easy to understand and complete. Most respondents, however, felt the questions may be challenging to comprehend by persons with lower than tertiary education and for persons not working in the health sector. One suggested the need to have a low literacy version of the multidimensional assessment. A respondent noted:

‘Some questions there are kind of technical. There are some questions there that an ordinary man may not be able to understand. For somebody that is educated, the wordings would not be a problem because the questions are specific enough for educated persons, but a layman or someone that is not well educated, might not be able to read some of the wordings in that questionnaire. But for me, it was okay, I don’t have a problem with them.’

‘If the questionnaire is sent to people that are not in the healthcare system, they might find some aspect difficult to fill. For example, section two talks about medical health status and it is asking whether somebody has arthritis, depression, herpes simplex. These are medical terminology. Somebody who is not in health sector might not know what these terminologies are and not know if they have the condition.’

‘It (the survey) is expected to be applicable to all persons. But when we look at the level of literacy of those affected by the pandemic, their level of literacy cannot be the same. Some people cannot even understand the questions. So I think, maybe, if there is a way, the questionnaire can be written in Pidgin, because some may not be able to read it in English, some may read and not understand. I think if this done it can be simplified to enable those with lower educational qualification to be able to fill it.’

One respondent had challenges with questions reflecting on sex and gender

‘Question 18 asked about sex, and 19 is still asking about gender. I was confused. Is gender and sex not the same thing? So I found that aspect difficult to complete. I don’t know if somebody can be born as a male and after sometimes, change to a woman.’

Most respondents felt the questionnaire was comprehensive as it covered basic issues related to COVID-19 mental health, and wellness of adults which are the focus of the study.

‘very comprehensive, very comprehensive … .. especially section 3, that is the pandemic stress index, it is very comprehensive, the options given in that session are okay, it covers every aspect.’

‘Very very comprehensive, because it touched many aspects. Even during the pandemic, it talked about the lockdown and many other things’

A respondent, however, noted that the enquiry on dental health was too limited and could be made more comprehensive. Another was interested in having the questionnaire explore the impact of the pandemic on tuberculosis like it did for HIV, and a third felt the question should explore about the impact of stigma facing persons who were positive to COVID-19 infection.

‘Sure it is comprehensive enough, but one thing I noticed is that there is a question that talks about dental... I think dental care or something like that, I just realized that it is just one or two questions of all the questions so I was like maybe that dental question just occurred to the writer and decided to just put it because I was also expecting more of dental health questions in the questionnaire but I think there is only one or two that addresses dental health.’

‘One aspect that I think should be added is about TB. When you look at TB and this COVID 19, you will see that they are related, they have a relationship. I believe with this COVID 19, a lot of TB cases will be missed because both have cough as their symptoms. When I look at it, I believe things about TB should be asked because when you look at it, you will see that so many questions were asked about HIV. I think it is very important that something about TB should be incorporated into the survey.’

‘What I would like to see added is that if somebody says he has it [COVID-19], maybe we asked the person when you were first informed that you were infected, what was your reaction? Maybe the questionnaire can explore the experience of stigma when infected: was he stigmatized by his family or by the community?’

‘Well, I can’t remember, if the researcher added location. I think adding location would have been a very good thing, to know how COVID-19 affects us geographically. I think questions like, what part of the country do you reside in could have been added’

## Discussion

There are no studies that validated an instrument to measure the multiple dimensions of mental health and wellness during the COVID-19 pandemic. There are instruments validated to measure fear of the pandemic with recommendation for future studies to assess diagnostic accuracy [46]. Some other instruments were also validated for use in screening, diagnosis, assessing the impact on and prognosis of mental health during the pandemic [47]. Other validated tools were limited in diversity of age, geographic coverage, COVID-19 exposure and socioeconomic status; or did not measure aspects of mental health like suicidal ideation or behavioral responses/ coping strategies [48]. The COVID-19 MEHEWE questionnaire was developed and validated to address some of these gaps. This study highlights several features of the COVID-19 MEHEWE questionnaire.

First, the overall content validity of the questionnaire was high and so were the values of all sections except for the items in section 2 assessing memory and cognitive status. We advise that these items be dropped when planning to use the MEHEWE questionnaire in the future. The validity of the MEHEWE study questionnaire is reinforced by the perceptions the participants in the qualitative part of the study. A respondent reiterated the need for the instrument to capture details on COVID-19 infection related stigma since this, like other forms of stigma, impacts negatively on the mental health and wellbeing of affected persons [49] and this was already included in the PSI section. There was a comment to measure the impact of COVID-19 infection on tuberculosis like HIV. The COVID-19 experience also impacts on the mental health and wellbeing of persons living with tuberculosis like it does for persons living with HIV [50]. The justification to further explore the link between the COVID-19 pandemic and oral health will need to be addressed in view of the emerging evidence about the relationship between COVID-19 infection and oral health [51].

Second, the Kappa and ICC showed different levels of test- retest reliability in different sections. The reliability of the sociodemographic profile and COVID-19 experience, MAC-Q, perceived social isolation, coping score and PLHIV was generally moderate. This maybe because some of these factors/ constructs change from time to time such as how participants reported their medical conditions including anxiety or dermatologic problems and how the pandemic impacted their relations with others. In these cases, the test retest scores would be low. By contrast, the PCL-C score had high reliability. These findings support the use of the items of the MEHEWE questionnaire in cross-sectional rather than longitudinal studies. Items with moderate reliability can be safely used to assess the prevalence and spread of the impact of the pandemic on health and wellbeing. However, their use to assess change in the same person regarding the impact of the pandemic should be done with caution due to the observed level of reliability. The high reliability of the PCL-C, on the other hand, supports its use for longitudinal studies.

Third, the Cronbach’s α showed excellent internal consistency for MAC-Q and PCL-C items and moderate or less consistency for the items assessing the quality of relations and coping strategies. This may be attributed to the few items used in these sections. For example, the coping scale had only three items. A balance is needed, however, between increasing the number of items to increase the internal consistency and the risk of greater respondent fatigue due to answering more questions.

Fourth, the PSI has not been previously validated. Using MCA, we showed that each of the behavior change items and the psychosocial impact items were not unidimensional. The first dimensions in each section explained most of the variance and had better internal consistency than the respective second dimensions. Also, the second dimension of the PSI behavior change included items describing change in status rather than changes in behavior that are done by individuals. This indicates that the PSI may need further revision and that it is not advised to use its items in their current state neither as unidimensional or multidimensional scales for which scores are calculated. Indeed, several items had low mean discrimination measures across dimensions or had conflicting discrimination in the two dimensions. Alternatively, the components of the items can be used individually without calculating overall or sub scores since they all had good content validity. The same applies to the items of the eleven self-care strategies which had moderate overall internal consistency and much higher internal consistency of dimension 1items. Further revision of these items is need possibly to remove or replace items so that the conflicting discrimination of items such as gardening and learning new skills is improved. The items of the PSI and the self-care strategies were specifically developed for use in relation to the COVID-19 pandemic. Other items such as those assessing coping and the PCL-C existed before the pandemic and were previously validated. Further studies improving the metrics of the PSI and self-care strategies may enable the assessment of other types of validity – such as construct and criterion validity- which were not possible in this study because of the modest performance of PSI and self-care strategies items.

The COVID-19 MEHEWE questionnaire would prove very useful for use during the pandemic, and can be adapted for use in future epidemics and pandemics of similar nature. The pandemic has ubiquitous effect on mental health with variable impact for different populations [52]. This questionnaire not only captures the possible multidimensional impact of the pandemic on mental health, but also includes parameters that can capture the experiences of subgroups that may be neglected like people living with HIV. The pandemic seems to affect the mental health and wellness of different populations in different ways as studies in Nigeria [53–55], and other global studies indicate [56, 57].

One of the strengths of the study is its methodology. The reliability and content validity of the instrument was determined by gathering quantitative and qualitative evidence. These different methodologies support the conclusions we drew about the validity and reliability of the various sections of the questionnaire and the recommendations made based on the findings. The large global sample used to assess the questionnaire is also a strength of the study. Though the study participants are drawn from different regions, not all countries are adequately represented. Country-level validation of the instrument is still needed for future use. Also, though there were respondents who used the Arabic, French, Spanish and Portuguese versions of the instrument, this does not preclude the need to validate these versions of the instruments. The instrument was also used in adults and assessing its validity and reliability in adolescents and young adults is needed. The need for population specific validation is strengthened by the observation made by the participants in the qualitative study that identified the instrument may be difficult to use for low-literate populations. A low literate edition of the multidimensional assessment may need to be developed. This instrument is reliable and valid enough to be adapted for that purpose.

One of the limitations of the study was our inability to assess the criterion validity and construct validity of the PSI since we were not able to calculate a score from its items. The multiple dimension and conflicting discrimination measures called for revision of the items and made the calculation of an overall score incorrect. In this present study, we combined responses across languages to assess the overall content validity. However, there may be differences by language in the content validity and/ or reliability of various items and because of this, future studies among subgroups with similar languages are needed. This language-specific evaluation should follow the changes recommended to improve the various metrics of the items by addition, deletion or modification.

## Conclusion

The tool used for the MEHEWE study for assessing the impact of the COVID-19 pandemic on mental health and wellness has good content validity and various levels of reliability that differed by section. It is a valid instrument to measure the impact of the COVID-19 pandemic in global cross-sectional studies. Some items need to be modified and others need to be omitted. The instrument needs to be validated in adolescents and young populations, in specific countries and in specific languages.

## Supplementary Information

**Additional file 1.**

**Additional file 2.**

## Data Availability

The dataset analysed in the current study is not publicly available because it is used for other publications at the present time but is available from the corresponding author on reasonable request.

## Abbreviations

CRISIS: Coronavirus Health Impact Survey
CVI: Content validity index
HIV: Human immuno deficiency virus
ICC: Intraclass correlation coefficient
MAC-Q: Memory assessment complaint questionnaire
MACS: Multi-center AIDS Cohort Study
MCA: Multiple correspondence analysis
MEHEWE: Mental health and wellbeing
PCL-C: PTSD CheckList – Civilian Version
PLHIV: People living with HIV
PSI: Pandemic stress index
PTSD: Post-traumatic stress disorder
WIHS: Women’s Interagency HIV Study
