# Supplementary material for: Validation of a COVID-19 mental health and wellness survey questionnaire
Source: BMC Public Health. 2022 Aug 8;22:1509. doi: 10.1186/s12889-022-13825-2 (PMC9358641; doi:10.1186/s12889-022-13825-2)
Supplement: Supplementary file 2 — Additional file 2. [file 12889_2022_13825_MOESM2_ESM.docx]

# Responses by country

| Country | N | % |
| --- | --- | --- |
| Afghanistan | 4 | <0.1 |
| Albania | 4 | <0.1 |
| Algeria | 14 | 0.1 |
| Andorra | 2 | <0.1 |
| Angola | 3 | <0.1 |
| Antigua and Barbuda | 2 | <0.1 |
| Argentina | 572 | 2.7 |
| Armenia | 5 | <0.1 |
| Australia | 54 | 0.3 |
| Austria | 8 | <0.1 |
| Azerbaijan | 7 | <0.1 |
| Bahamas | 1 | <0.1 |
| Bahrain | 16 | 0.1 |
| Bangladesh | 18 | 0.1 |
| Barbados | 1 | <0.1 |
| Belarus | 18 | 0.1 |
| Belgium | 11 | 0.1 |
| Benin | 13 | 0.1 |
| Bhutan | 1 | <0.1 |
| Bolivia | 3 | <0.1 |
| Bosnia and Herzegovina | 250 | 1.2 |
| Botswana | 28 | 0.1 |
| Brazil | 110 | 0.5 |
| Bulgaria | 3 | <0.1 |
| Burkina Faso | 3 | <0.1 |
| Cambodia | 5 | <0.1 |
| Cameroon | 12 | 0.1 |
| Canada | 159 | 0.8 |
| Chile | 148 | 0.7 |
| China | 35 | 0.2 |
| Colombia | 63 | 0.3 |
| Congo, Democratic Republic of the | 5 | <0.1 |
| Congo, Republic of the | 20 | 0.1 |
| Cote d'Ivoire | 32 | 0.2 |
| Croatia | 1 | <0.1 |
| Cuba | 1 | <0.1 |
| Cyprus | 5 | <0.1 |
| Czechia | 5 | <0.1 |
| Denmark | 5 | <0.1 |
| Dominica | 1 | <0.1 |
| Dominican Republic | 1 | <0.1 |
| Ecuador | 15 | 0.1 |
| Egypt | 760 | 3.6 |
| Estonia | 2 | <0.1 |
| Eswatini (formerly Swaziland) | 5 | <0.1 |
| Ethiopia | 7 | <0.1 |
| Finland | 458 | 2.2 |
| France | 31 | 0.1 |
| Gambia | 9 | <0.1 |
| Georgia | 2 | <0.1 |
| Germany | 55 | 0.3 |
| Ghana | 364 | 1.7 |
| Greece | 15 | 0.1 |
| Haiti | 1 | <0.1 |
| Honduras | 1 | <0.1 |
| Hungary | 213 | 1.0 |
| Iceland | 3 | <0.1 |
| India | 765 | 3.6 |
| Indonesia | 63 | 0.3 |
| Iran | 17 | 0.1 |
| Iraq | 10 | <0.1 |
| Ireland | 24 | 0.1 |
| Israel | 14 | 0.1 |
| Italy | 18 | 0.1 |
| Japan | 8 | <0.1 |
| Jordan | 713 | 3.4 |
| Kazakhstan | 6 | <0.1 |
| Kenya | 38 | 0.2 |
| Kosovo | 11 | 0.1 |
| Kuwait | 167 | 0.8 |
| Laos | 2 | <0.1 |
| Lebanon | 11 | 0.1 |
| Lesotho | 2 | <0.1 |
| Liberia | 18 | 0.1 |
| Libya | 14 | 0.1 |
| Lithuania | 31 | 0.1 |
| Luxembourg | 1 | <0.1 |
| Malawi | 20 | 0.1 |
| Malaysia | 17 | 0.1 |
| Maldives | 1 | <0.1 |
| Mali | 32 | 0.2 |
| Malta | 1 | <0.1 |
| Marshall Islands | 1 | <0.1 |
| Mauritania | 1 | <0.1 |
| Mauritius | 7 | <0.1 |
| Mexico | 627 | 3.0 |
| Monaco | 1 | <0.1 |
| Mongolia | 5 | <0.1 |
| Morocco | 8 | <0.1 |
| Myanmar (formerly Burma) | 7 | <0.1 |
| Namibia | 22 | 0.1 |
| Nepal | 4 | <0.1 |
| Netherlands | 45 | 0.2 |
| New Zealand | 19 | 0.1 |
| Nicaragua | 1 | <0.1 |
| Nigeria | 4596 | 21.8 |
| North Macedonia (formerly Macedonia) | 1 | <0.1 |
| Norway | 4 | <0.1 |
| Oman | 5 | <0.1 |
| Pakistan | 1689 | 8.0 |
| Palau | 1 | <0.1 |
| Palestine | 38 | 0.2 |
| Panama | 1 | <0.1 |
| Paraguay | 7 | <0.1 |
| Peru | 122 | 0.6 |
| Philippines | 529 | 2.5 |
| Poland | 5 | <0.1 |
| Portugal | 19 | 0.1 |
| Qatar | 26 | 0.1 |
| Romania | 7 | <0.1 |
| Russia | 10 | <0.1 |
| Rwanda | 15 | 0.1 |
| Saint Lucia | 1 | <0.1 |
| San Marino | 1 | <0.1 |
| Saudi Arabia | 958 | 4.5 |
| Senegal | 10 | <0.1 |
| Serbia | 38 | 0.2 |
| Sierra Leone | 7 | <0.1 |
| Singapore | 3 | <0.1 |
| Slovenia | 4 | <0.1 |
| Solomon Islands | 1 | <0.1 |
| South Africa | 573 | 2.7 |
| South Korea | 6 | <0.1 |
| South Sudan | 4 | <0.1 |
| Spain | 38 | 0.2 |
| Sri Lanka | 3 | <0.1 |
| Sudan | 221 | 1.0 |
| Sweden | 16 | 0.1 |
| Switzerland | 9 | <0.1 |
| Syria | 571 | 2.7 |
| Taiwan | 53 | 0.3 |
| Tanzania | 21 | 0.1 |
| Thailand | 37 | 0.2 |
| Togo | 1 | <0.1 |
| Trinidad and Tobago | 1 | <0.1 |
| Tunisia | 3 | <0.1 |
| Turkey | 78 | 0.4 |
| Uganda | 65 | 0.3 |
| Ukraine | 12 | 0.1 |
| United Arab Emirates (UAE) | 147 | 0.7 |
| United Kingdom (UK) | 752 | 3.6 |
| United States of America (USA) | 753 | 3.6 |
| Uruguay | 3 | <0.1 |
| Uzbekistan | 1 | <0.1 |
| Venezuela | 54 | 0.3 |
| Vietnam | 6 | <0.1 |
| Yemen | 332 | 1.6 |
| Zambia | 4 | <0.1 |
| Zimbabwe | 46 | 0.2 |
| Samao - American | 1 | <0.1 |
| Western Sahara | 1 | <0.1 |
| Yugoslavia | 1 | <0.1 |
| Total | 18253 | 86.5 |
| Unidentified | 2853 | 13.5 |
| Grand total | 21106 | 100.0 |
